# Refining mechanistic models of hallucinations for enhanced translatability

**DOI:** 10.1038/s41398-025-03773-x

**Published:** 2025-12-03

**Authors:** Justin Buck, Kiyohito Iigaya, Guillermo Horga

**Affiliations:** 1 Department of Psychiatry, Columbia University, New York, NY USA; 2 Department of Neuroscience, Columbia University, New York, NY USA; 3 Department of Psychiatry, New York State Psychiatric Institute, New York, NY USA

**Keywords:** Schizophrenia, Human behaviour

## Abstract

Over the past two decades, foundational work has provided key insights into the cognitive and neural basis of hallucinations. This progress has led to the development of several families of theories, each of which propose that hallucinations arise from distinct cognitive mechanisms. Since these cognitive mechanisms likely map onto separate circuit-level implementations, arbitrating between them is critical to advance our understanding of hallucination pathophysiology and guide the development of novel targeted therapeutics. However, several obstacles have hindered this progress, including the under-specification of theories and inadequate comparative testing. To overcome these challenges, and following best practices in cognitive computational neuroscience, theories should **1)** articulate computational and biological details at a level that allows the generation of precise, testable predictions, and **2)** be evaluated using experiments designed to emphasize their unique signatures to facilitate falsification. To illustrate this general approach, we demonstrate how theory-driven computational models constrained by well-replicated findings across basic, preclinical, and clinical neuroscience can provide a principled means to prioritize falsifiable mechanistic theories of auditory hallucinations. We then discuss how these models can be used to inform the development of richer behavioral paradigms and analytical approaches that enable direct comparisons between competing theories. Overall, we propose a general strategy for the specification and falsification of candidate mechanisms underlying (auditory) hallucinations – a critical prerequisite for refining hallucination theories and advancing their translational potential.

## Introduction

Mechanistic theories are an invaluable tool for clinical translation. By specifying how core features of hallucinations arise, such models can guide the identification of neurobiological treatment targets. Several promising mechanistic theories of hallucinations are currently under investigation in the field [1–13]. Arbitrating between these theories is critically important for translation because they likely map onto distinct neurobiological substrates, each of which could represent a distinct treatment target [14]. Progress in this direction requires theories that are described with enough detail to be rigorously tested by experiments that are designed to facilitate falsification [15–17].

A historical example illustrates potential barriers to achieving this goal. In 1543, Nicolaus Copernicus proposed a *heliocentric* (sun-centered) model of the solar system, challenging the dominant *geocentric* (earth-centered) model. This new model offered simpler explanations for puzzling phenomena like retrograde planetary motion, but lacked key details, which led to worse quantitative predictions than the geocentric model – which had been finely tuned to fit observations. Progress required both refinement of the heliocentric model and the development of decisive tests. In the 17th century, Johannes Kepler’s discovery of elliptical orbits and Isaac Newton’s laws of motion and gravity transformed the heliocentric model, enabling more accurate predictions and the generation of new, testable hypotheses. Crucially, experiments that directly pitted the two theories against each other on a level playing field (e.g., Galileo’s observation of the phases of Venus), provided compelling evidence for the heliocentric model, ultimately leading to its universal acceptance.

Similarly, despite important advances in theoretical accounts of hallucinations [1–4, 7, 11, 13], we argue that the field would benefit from models with greater specificity and mechanistic detail that can generate more concrete, testable cognitive and biological predictions. Then, experiments designed to separate these predictions could be used to refine and rule out models [16]. In this vein, here we build from seminal research in the computational psychiatry of hallucinations [2, 4, 7, 14, 15] and apply established best practices in cognitive/computational neuroscience [16, 18] to develop a pipeline for the description, prioritization, and testing of hallucination theories.

## Theories of hallucinations

Two families of theories have been particularly influential in the mechanistic study of hallucinations: *predictive-processing* and *salience-based* theories.

Predictive-processing theories of hallucinations posit that hallucinations arise from alterations in perceptual inference [3, 6, 11, 13, 19–21]. One prominent instantiation, the ‘strong-prior’ hypothesis, proposes that sensory expectations are overweighted relative to sensory evidence, which leads to false perceptions [1, 6]. In general, these theories emphasize the role of environmental uncertainty in shaping expectations [22, 23] and dopamine’s role in signaling this uncertainty [24–27]. These theories are supported by evidence of altered statistical learning in patients with schizophrenia [20, 21, 28] and have been formalized using Bayesian models of perceptual inference [3, 6].

Salience-based (also called ‘aberrant salience’) theories posit that hallucinations arise from altered attribution of significance to irrelevant or random stimuli [7, 8, 29, 30]. These theories were originally inspired by the therapeutic efficacy of antidopaminergic drugs [31] and seminal research on dopamine’s role in attributing motivational salience [32], but have evolved to incorporate a wide variety mechanisms. Among these is dopamine’s role in signaling reward prediction errors (rPEs) [33–35], through which aberrant learning of stimulus value could indirectly distort perceptual experience [2, 30, 36]. Broadly consistent with this perspective, alterations in reward processing have been identified in patients with schizophrenia [2, 19, 30, 37, 38], although specific relationships with hallucinations are less clear. Attempts have also been made to incorporate salience into predictive processing perspectives through related principles like surprise [3, 28], but specific neural computations and their biological implementation are still unclear.

**Fig. 1:**
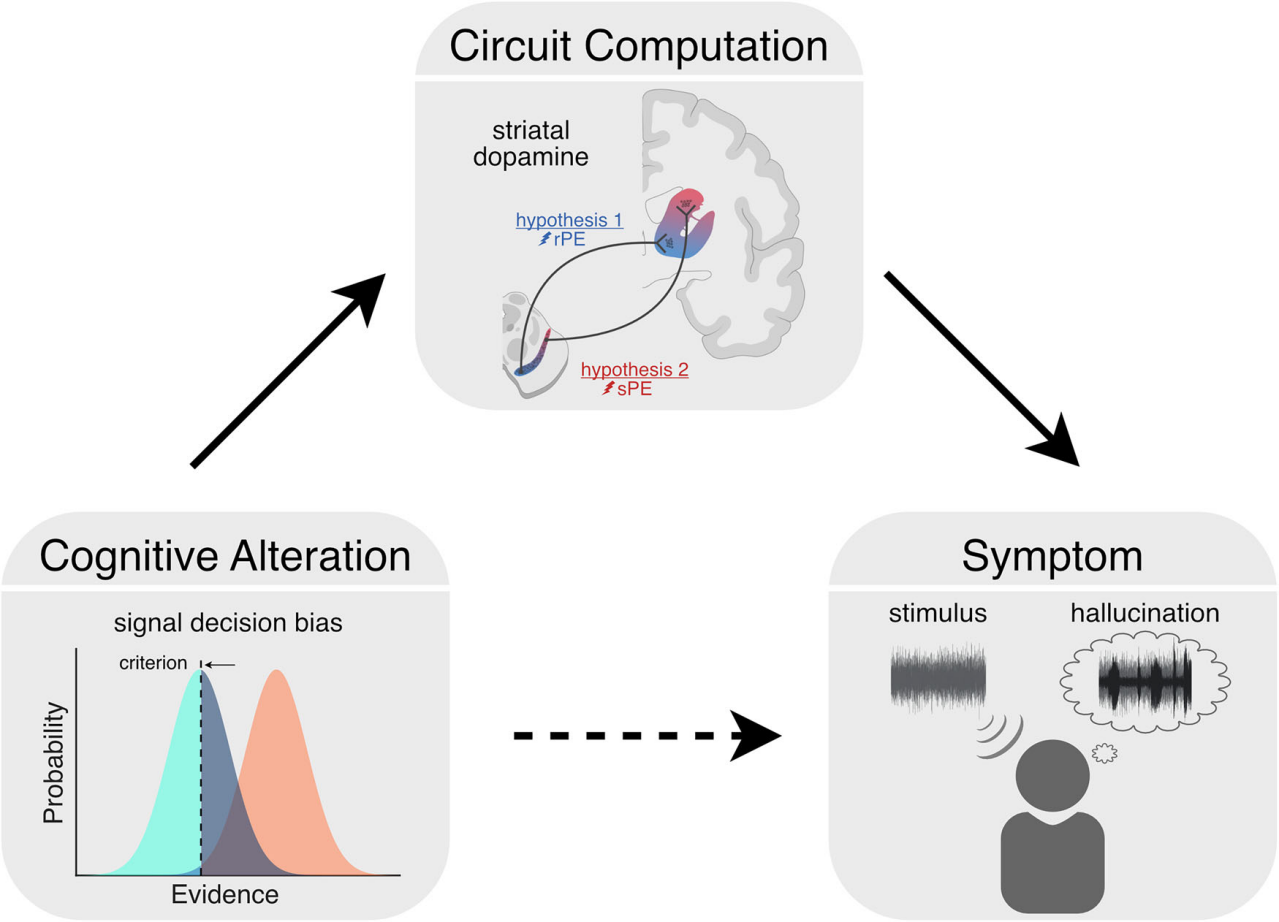
Bridging changes in cognition and psychiatric symptoms with explicit circuit mechanisms. Translatable theories should explain how a cognitive process is implemented by a biological circuit and how alterations in this mechanism drive altered computations and the symptom of interest. For example, extensive evidence has linked both excess striatal dopamine transmission and shifted decision criteria to the development and level of hallucinations. However, the underlying cognitive and circuit mechanisms remain unclear. Theories that propose circuit mechanisms mediating the relationship between excess striatal dopamine, biased decision criteria, and hallucinations are essential for translation. For example, excess mesostriatal or nigrostriatal dopamine may correspond to altered reward prediction error (rPE) or sensory prediction error (sPE) signaling, respectively. Both options could bias expectation learning and influence perception but would represent distinct treatment targets.

A translatable mechanistic hypothesis should specify how an altered circuit computation leads to hallucinations [14] (Fig. 1). In other words, the hypothesis should explain how false perceptions arise from an altered cognitive process implemented by a specific biological substrate. Do these hypotheses meet this standard? The ‘strong-prior’ hypothesis is often described in relatively concrete mathematical terms but typically lacks a precise biological implementation (including in our own previous work [1]). The ‘aberrant salience’ hypothesis specifies striatal dopamine as a key biological substrate but its cognitive/computational basis has largely been described in abstract terms (but see [2]). Thus, both theories require further development to make precise cognitive and neural predictions that are crucial for translation to clinical applications.

Additionally, these hypotheses have predominantly been tested using separate experimental frameworks. While the ‘strong-prior’ hypothesis is often evaluated using sensory expectation paradigms [20, 21, 39–41], the ‘aberrant salience’ hypothesis is often tested using reward learning tasks [29, 37, 38, 42]. These divergent methods make it difficult to determine whether empirical results reflect the validity of a theory or limitations of an experimental design. To fairly evaluate these and other viable theories, a unified experiment that directly pits these theories against each other would be ideal. Such an experiment would emphasize the qualitative differences between the theories, promoting falsification and refinement.

In sum, we see room for progress in the description and testing of mechanistic theories of hallucinations. In what follows, we endeavor to illustrate a general strategy by which detailed mechanistic hypotheses can be developed and tested with experiments that evaluate them on a level playing field.

## Constraining a mechanistic theory of hallucinations

We aim to develop theory-driven computational models of hallucinations grounded in fundamental neural and cognitive mechanisms. Specifically, we focus on generative models that explain how intraindividual and interindividual variability in hallucinations arises. These models can be empirically validated by testing whether the behavioral signatures they predict, such as patterns of misperceptions in a cognitive task, systematically relate to hallucinations. In the context of this paper, we are interested in models that account for variability in hallucination level, reflecting the overall intensity and frequency of hallucinations. These models are not intended to account for the specific content of hallucinations.

**Fig. 2:**
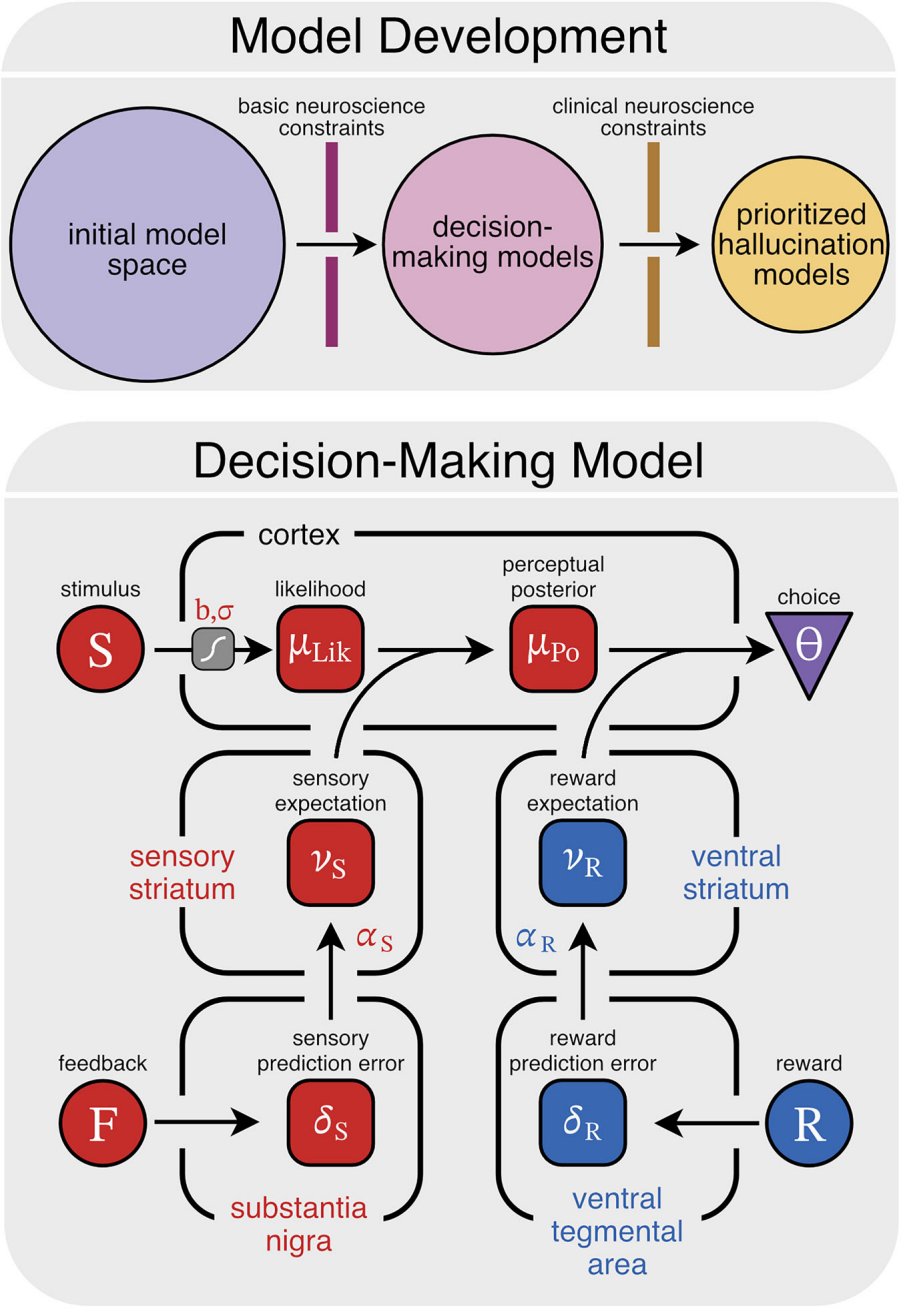
Operationalizing mechanistic theories of hallucinations using computational modeling. We will use a two-step process to specify detailed models of hallucinations (top). First, we will apply constraints from the basic neuroscience literature (group A) to build a plausible decision-making model. Second, we will use constraints from the human clinical literature (group B) to prioritize candidate models of hallucinations. Group A constraints led to the development of a decision-making model that updates sensory and reward expectations based on experience and integrates these expectations with current sensory evidence to determine if a signal was present or absent on each trial (bottom).

To achieve this goal, we follow a two-step approach (Fig. 2). First, we will draw on established findings in cognitive neuroscience to construct a general computational model. This model will specify key details about relevant cognitive mechanisms and their circuit implementation. Second, we will evaluate whether specific alterations in elements of this general model capture behavioral patterns robustly linked to hallucinations. We will refer to empirical findings we use to narrow the space of potential models as *constraints* [43] (Table 1).

**Table 1:** Empirical constraints on decision-making and hallucination models.

Constraint Type	Decision-Making Model Constraints	Hallucination Model Constraints
Behavioral	**Choice and confidence are sensitive to:** A1. Reward and stimulus history A2. Choice difficulty	**Hallucinations scale with:** B1. Signal decision bias B2. False alarm confidence
Neural	**Striatal dopamine encodes:** A3. Teaching (e.g., prediction-error) signals A4. Anatomically distinct information	**Hallucinations scale with:** B3. Striatal dopamine transmission

### Constraints from the basic neuroscience literature

Hallucinations are internal perceptual experiences that occur in the absence of corresponding external stimuli, and their subjective experience is often comparable to true percepts in terms of their phenomenological (e.g., acoustic) features [44]. Furthermore, substantial evidence indicates that perceptual disturbances, including hallucinations, exist on a continuum across the general population and clinical groups [13, 21, 45–48]. Together, these results support the building of hallucination theories upon basic theories of perceptual decision-making [6, 49].

Perceptual decisions are often made under uncertainty. For example, in a subway station, a sound that resembles your name being called out may be obscured by ambient noise. You can resolve this uncertainty by incorporating your learned expectations to decide if your name was actually called [22, 50]. Specifically, how likely it is that someone would call your name (i.e., sensory expectations) and the desirability of concluding your name was called (i.e., reward expectations). Empirically, expectations (often inferred from sensory and reward histories [13, 24, 51, 52]) bias choices (Constraint A.1). If you were planning to meet someone, both expectations could bias you toward deciding that your name was called, even if no one called your name. Since reward expectations can bias decisions without reflecting a true percept, additional reports such as confidence are useful for ascertaining the determinants of a particular choice [53–55]. In decision-making tasks, confidence reports are also biased by expectations (Constraint A.1) and are commensurate with an observer’s subjective probability of being correct (i.e., confidence is higher on correct trials and this effect scales with stimulus difficulty [13, 51, 52]; Constraint A.2).

At the circuit level, expectation learning is thought to be facilitated by striatal dopamine (Constraints A.3). Specifically, *mesolimbic* dopamine signals reward prediction errors (rPEs) [33–35], which represent the difference between received and expected reward, prompting an update of reward expectations. More recent work in rodents suggests that *nigrostriatal* dopamine encodes distinct, reward-independent information [56, 57] relevant to perception [13, 58, 59]. Consistently, the human striatum is also sensitive to sensory statistics [60] and involved in sensory learning [61]. While the precise computation instantiated by nigrostriatal dopamine is still an area of active research, this evidence supports constraining reward and sensory processing to distinct dopaminergic circuits [13, 56–58, 62–64] (Constraint A.4; but see [65–68]). Overall, these data support a model whereby dopamine-mediated prediction errors in distinct pathways are used to learn stimulus probability (i.e., how likely it was that my name was called) and decision value (i.e., the desirability of concluding my name was called) which are incorporated into decisions and confidence (Fig. 2).

### Constraints from the clinical neuroscience literature

In the example above, incorrectly concluding your name was called would in principle be analogous to a hallucination – the two have interchangeable definitions as perceptual reports of a stimulus in its objective absence. Consistent with this conceptual link, higher hallucination propensity correlates with increased reports of experiencing stimuli in signal detection tasks (i.e., false alarms and hits; Constraint B.1) and higher confidence in these decisions (Constraint B.2) in both patients with hallucinations [21, 46, 69] and the general population [13, 46, 48, 70, 71] (Figure S1). Importantly, not all false alarms reflect true hallucinations. However, *high-confidence* false alarms are thought to capture them because they represent a deliberate commitment to reporting a genuine percept, consistent with the typical conviction of experienced hallucinations in psychosis. These results are well-replicated across clinical and non-clinical populations, research groups, and methodological variations, suggesting that a mechanistic explanation for increased high-confidence false alarms is a key ingredient for any computational model of hallucinations. Note that we focus here on auditory hallucinations given their relevance to idiopathic psychotic disorders, although similar cognitive alterations in vision [39–41] suggest that our discussion will also be relevant to perceptual disturbances in other modalities.

At the neural level, converging evidence from multiple lines of work has implicated excess striatal dopamine in the development of hallucinations (reviewed in greater depth elsewhere [6]). Briefly, molecular neuroimaging studies measuring striatal dopamine function have repeatedly shown a specific positive relationship between striatal dopamine function (e.g., dopamine synthesis and release capacity) and the intensity of positive symptoms, including hallucinations [20, 72–76]. Consistently, pharmacological studies have demonstrated a dose-dependent relationship between pro-dopaminergic drugs and psychotic symptoms [77–80] and the therapeutic benefits of antidopaminergic drugs on psychotic symptoms are definitively established [31] and depend on striatal dopamine-receptor blockade [81]. Recent results from the preclinical literature have also causally implicated stimulation of sensory-striatal dopamine in high-confidence false perception [13].

This evidence has been taken to suggest that excess striatal dopamine is likely *sufficient* for the generation of hallucinations – even if it is not necessary [6, 82]. Indeed, other neurotransmitters, including glutamate [83–85] and acetylcholine [86–88], have also been implicated in hallucinations, but their mechanistic roles in perceptual decision-making and psychosis are much less clear. Here, we will use the extensive body of research into dopamine function to provide initial – and likely imperative – neurobiological constraints for hallucination theories. Specifically, viable theories should link increased striatal dopamine to elevated rates of high-confidence false alarms (Constraint B.3). We would argue that theories that do not satisfy these constraints (B.1-B.3) should be deprioritized by virtue of their limited explanatory power to account for critical empirical findings.

## Decision-making model

Having defined the relevant constraints, we will now proceed with the first step in the model development process: constructing a computational model of decision-making (Fig. 2). Computational models are particularly useful for formalizing and testing mechanistic hypotheses [89]. They offer simple and interpretable explanations for complex phenotypes and can generate specific predictions that can be compared against empirical data [89–91]. We do not intend to make strong claims about a specific model architecture but rather demonstrate how a reasonable decision-making model can be used as a scaffold for developing and testing theories of hallucinations.

### Perceptual inference

Consider the subway example where you are trying to decide whether your name was called. Following standard models of perception [6, 13] your percept ${\mu }_{{Po}}$ can be represented as a combination of the external sensory evidence (or likelihood ${\mu }_{{Lik}}$) and your internal sensory expectations ${\nu }_{S}$ (i.e., the prior probability that your name was called). We can write this as a sum of log-odds (or logits).

1 $${\rm{logit}}({\mu }_{{Po}})={\rm{logit}}\left({\nu }_{S}\right)+{\rm{logit}}\left({\mu }_{{Lik}}\right)$$

${\mu }_{{Lik}}$ is the internal representation of the stimulus $s$ and is drawn from a cumulative normal distribution $\phi$ with mean $b$ and standard deviation $\sigma$:

2 $${\mu }_{{Lik}}=\phi ({s;b},\sigma )$$

### Choice

Your decision $\theta$ is influenced by both your percept ${\mu }_{{Po}}$ and your reward expectation (i.e. the desirability of concluding your name was called). We represent reward expectation ${\nu }_{R}$ as the probability of a reward following a “Yes” choice ($\theta =1$), with the probability of reward for a no choice ($\theta =0$) being complementary. This simplifies the choice computation and enables more direct comparisons with sensory expectations due to the parallel structure. The decision with the larger expected value is made [22]:

3 $$\theta =\left\{\begin{array}{cc}1, & \mathrm{if}\,\left[\mathrm{logit}\left({\nu }_{R}\right)+\mathrm{logit}\left({\mu }_{{Po}}\right)\right]\ge 0\\ 0, & \mathrm{otherwise}\end{array}\right.$$

Note that this decision rule is equivalent to a signal detection framing in which sensory evidence is compared to a decision criterion that is informed by expectations (Supplemental Methods).

### Confidence

As we discussed above, behavioral reports like confidence can be used to dissociate the relative influences of perception and reward on decisions [53–55]. Following similar models [13, 22, 24], perceptual confidence ${C}_{P}$ is derived from perceptual belief ${\mu }_{{Po}}$ while reward confidence ${C}_{R}$ is derived from reward expectations ${\nu }_{R}$. Decision confidence ${C}_{D}$ integrates both into a single metric using a logit sum:

4 $${C}_{P}={\mu }_{{Po}}$$

5 $${C}_{R}=\,\left\{\begin{array}{cc}{\nu }_{R}, & {if\; \theta }=1\\ 1-{\nu }_{R}, & {if\; \theta }=0\end{array}\right.$$

6 $${\rm{logit}}\left({C}_{D}\right)={\rm{logit}}({C}_{P})+{\rm{logit}}\left({C}_{R}\right)$$

### Reward learning

So far, we have considered a single decision, but sensory and reward expectations are dynamic and are learned through experience with environment. Reward expectations are learned through reward prediction errors ${\delta }_{R}^{t}$ using the delta rule [33–36, 51, 92] at the current time point $t$ (Constraint A.3):

7 $${\delta }_{R}^{t}=\left({R}^{t}-{C}_{R}^{t}\right)$$

where ${R}^{t}$ is the reward outcome (i.e., the choice was or was not desirable). For simplicity, we will consider this to be a binary outcome. This reward prediction error is then scaled by a learning rate$\,{\alpha }_{R}$ and used to update your reward expectation:

8 $${\nu }_{R}^{t+1}=\left\{\begin{array}{l}{\nu }_{R}^{t}+{\alpha }_{R}\,\cdot \,{\delta }_{R}^{t},{if\; \theta }=1\\ {\nu }_{R}^{t}-{\alpha }_{R}\,\cdot \,{\delta }_{R}^{t},{if\; \theta }=0\end{array}\right.$$

### Sensory learning

Sensory expectations are learned through distinct error signals (Constraint A.4). Sensory prediction errors ${\delta }_{S}^{t}$ are computed using a delta rule [13]:

9 $${\delta }_{S}^{t}=\left({F}^{t}-{C}_{P}^{t}\right)$$

where ${F}^{t}$ is sensory feedback (i.e., your name was or was not called). For simplicity, we will consider a case where veridical feedback is available but more sophisticated learning rules can operate without feedback [93]. This sensory prediction error is then scaled by a learning rate $\,{\alpha }_{S}$ and used to update the observer’s sensory expectation ${\nu }_{S}$:

10 $${\nu }_{S}^{t+1}={\nu }_{S}^{t}+{\alpha }_{S}\,\cdot \,{\delta }_{S}^{t}$$

Each time step, sensory and reward expectations will shift in the direction of the true outcome (i.e., sensory feedback or reward) and the magnitude of the shift will depend on how unexpected the outcome was.

Overall, the model uses learned expectations (${\nu }_{S}$ and ${\nu }_{R}$) and sensory evidence (${\mu }_{{Lik}})$ to decide if a signal was present or absent on each trial. This structure results in choices and confidence that are sensitive to stimulus and reward history (Constraint A.1) and the degree of sensory evidence (Constraint A.2) which can be seen in simulated choice and confidence reports (Figs. S2–3).

## Prioritizing hallucination models

Having defined a plausible decision-making model, we now proceed to the second step of the model development process: identifying alterations in the model parameters that reproduce hallucination-related behavioral patterns (Constraints B.1 & B.2; Fig. 2). Rather than exhaustively outlining all potential models, we illustrate how a constraint-based strategy can be used to prioritize the most promising candidates, inspired by similar efforts in other fields like genomics [94].

### Assessing candidate models

**Fig. 3:**
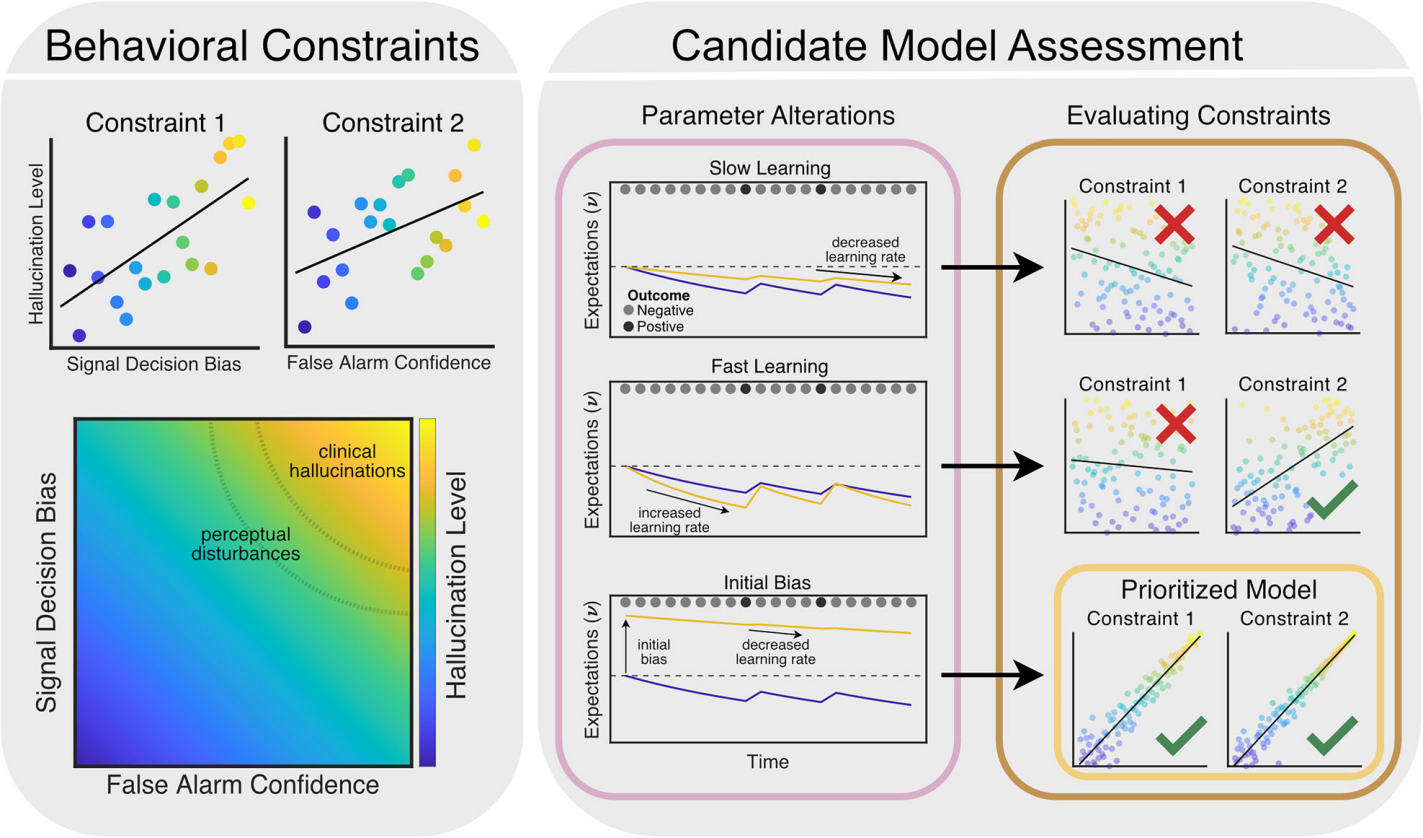
Prioritizing candidate hallucination model variants. Candidate models of hallucinations can be prioritized by their ability to account for well-established empirical findings. Given the robust relationship between hallucination propensity and increased signal decision bias and false alarm confidence in signal detection tasks, we expect a candidate alteration to also drive these behaviors (left). To evaluate these criteria, we simulated observers across a range of parameter alteration magnitudes. If both signal decision bias and false alarm confidence scale with a parameter alteration magnitude, we prioritize that model. For example, we find that as learning rate decreases, (i.e., slow learning; Eq. 8 & 10) neither signal decision bias nor false alarm confidence increase, so this model is not prioritized. In contrast, if a reduced learning rate is combined with a pre-existing bias (i.e., initial bias), both signal decision bias and false alarm confidence increase.

To evaluate a candidate model, we simulate behavior on a signal detection task (see Supplemental Methods). We consider parameter alterations that could plausibly arise from excess striatal dopamine (Constraint B.3) and conclude that an alteration meets prioritization criteria if it increases both signal decision bias (Constraint B.1) and false alarm confidence (Constraint B.2; Fig. 3, S4). As illustrative test cases, we focus on models corresponding to the ‘strong-prior’ and ‘aberrant salience’ hypotheses.

#### ‘Strong prior’

The ‘strong prior’ hypothesis proposes that increased prior weighting biases perception away from sensory evidence, leading to hallucinations. A strong prior is in principle analogous to a reduced learning rate in our model (i.e., smaller $\alpha$; see Supplemental Methods Eqs. S5–S7) because the learning rate governs the degree to which new evidence is used to update beliefs. Therefore observers with low learning rates maintain their prior beliefs even when faced with contradictory evidence. We find that models with slow sensory or reward learning did not pass our prioritization criteria (Fig. 3; Table 2).

However, we note that patients with hallucinations display increased signal decisions in auditory detection tasks with stable statistics (e.g., probability of signal is maintained at 0.5 throughout the task [13, 46, 48, 69]) and voice hearers show increased ability to detect corrupted speech without prior exposure [95]. These findings suggest that a candidate alteration could reflect a pre-existing bias (or a prior acquired based on experiences outside the task) which can be operationalized in our framework by biasing initial sensory expectations (${\nu }_{S}^{t=0} > \,0.5$). But if an observer with such initial bias learns normally, their expectations would adjust over time, inconsistent with empirical data. When biased initial sensory expectations are combined with a reduced sensory learning rate (consistent with a type of pre-existing ‘strong prior’), these observers maintain their biased sensory expectations despite contradictory evidence. Models with an initial bias and reduced learning in either the sensory or reward domains satisfy our prioritization criteria (Fig. 3; Table 2). While the ‘strong prior’ hypothesis does not necessarily prescribe an initial bias in expectations [1], such a bias is nonetheless consistent with this hypothesis and seems necessary to account for empirical data.

**Table 2:** Evaluation of potential models according to group B constraints.

Model	Cognitive Evidence	Link to Dopamine (B.3)	Parameter Alteration		B.1	B.2	Priority?
Sensory Noise	Altered sensory processing in SCZ [110, 111]	DA can modulate cortical noise [112]	↑ *σ*		×	✓	No
Fast Learning	Increased learning for irrelevant stimuli in SCZ [30] and in PD patients given DA agonists [113]	DA involved in signaling decision uncertainty in animals [27, 52] and humans [25, 26]	S	↑ *α*_*S*_	×	✓	No
			R	↑ *α*_*R*_	×	✓	No
Slow Learning	Decreased statistical learning in SCZ [1, 6, 20, 21]	Decreased learning correlates with striatal DA release and hallucination level [20]	S	↓ *α*_*S*_	×	×	No
			R	↓ *α*_*R*_	×	×	No
Initial Bias	Baseline signal decision bias correlates with hallucination proneness [46, 48, 69] and improved detection of corrupted speech in NCVH [95]	DA agonism induces Go bias and reduces learning from positive outcomes [114]	S	↑${\nu }_{S}^{t=0}$↓ ${\alpha }_{S}$	✓	✓	Yes
			R	↑${\nu }_{R}^{t=0}$↓ ${\alpha }_{R}$	✓	✓	Yes
Asymmetric Learning	Learning asymmetries linked to striatal DA genotypes [96] and DA agonism in PD [97, 98]	Models of increased tonic dopamine release can drive asymmetric learning [115]	S	${\alpha }_{S}^{F=1} > \,{\alpha }_{S}^{F=0}$	✓	✓	Yes
			R	${\alpha }_{R}^{R=1} > \,{\alpha }_{R}^{R=0}$	×	✓	No
Hybrid Bias	Altered learning of stimulus-reward associations in SCZ [30] and reward schedules influence sensory processing [59, 102]	DA can represent complex combinations of variables [103–105] (e.g., threatening stimuli [57])	↑${{\alpha}^{F=1\,\cap {R}=1}}$		✓	✓	Yes

See Figure S4 for group B constraint evaluation simulations.

*SCZ* schizophrenia, *DA* dopamine, *NCVH* non-clinical voice-hearer, *PD* Parkinson’s disease, *S* sensory, *R* reward.

#### ‘Aberrant salience’

Reward learning-based implementations of the ‘aberrant salience’ hypothesis propose that striatal dopamine indirectly alters perception by disrupting the learning of the value associated with stimuli through changes in rPEs [30, 36]. This hypothesis has previously been operationalized by assuming that positive reward prediction errors are of larger absolute magnitude than their negative counterparts in psychosis ($\left|{\delta }_{R}^{+}\right| > \left|{\delta }_{R}^{-}\right|$) [2, 36]. Consistent with this notion, genotypes known to influence striatal dopamine [96] and pharmacological dopamine agonism [97, 98] have been linked to asymmetric learning biases in humans. This mechanistic interpretation can be approximated in our modeling framework with biased learning from positive reward predictions (${\alpha }_{R}^{+} > \,{\alpha }_{R}^{-}$). This model does not pass our prioritization criteria (Table 2). In contrast, a model with biased learning from sensory predictions (${\alpha }_{S}^{+} > \,{\alpha }_{S}^{-}$) does pass or prioritization criteria. However, since this model would develop exaggerated sensory expectations, it relates more closely to predictive-processing theories.

In general, our theoretical results support deprioritizing models that foreground standard value learning rPE-based mechanisms. Consistent with this, previous empirical attempts have generally failed to produce evidence linking altered value learning rPE signals and hallucinations [2, 30, 38, 99, 100]. That said, models that propose a more nuanced role for rPEs, or those focused on cortical gating mechanisms [2], could still be considered. For example, the value associated with certain environmental states (e.g., signal presence versus absence) can bias attentional [101] or sensory processes [59, 65–67, 102] to influence perceptual decisions. Additionally, dopamine signals may represent complex combinations of reward and sensory features [57, 103–105] (e.g., threat prediction-errors [106]), so models of hallucinations that consider reward-sensory crosstalk may deserve further consideration. Speaking to this, one possible model that satisfies our prioritization criteria learns more rapidly when signal trials are rewarded (hybrid bias model; Table 2).

So far, we have illustrated a strategy to develop and prioritize biologically and computationally detailed models of hallucinations (Table 2). This tentative taxonomy relates to, but does not map perfectly onto, prevalent hypotheses: only certain concrete operationalizations of a given hypothesis satisfy our a priori criteria. We believe that this exercise thus highlights the importance of concretely specifying generative models and comparing their predictions against empirical data.

## Testing prioritized models

Now that we have prioritized a set of candidate models, we will evaluate whether they can be empirically distinguished in a hypothetical experiment. This is a nontrivial challenge because all prioritized models have shared behavioral patterns (Constraints B.2 & B.3). As with comparisons between heliocentric and geocentric models, the most compelling tests elicit qualitatively distinct signatures from each candidate model.

Here, we focus on signal detection tasks due to the robust relationships previously identified with hallucination level. However, in principle, other tasks that probe sensory and reward learning (e.g., conditioning paradigms) could also be considered as long as they permit reliable estimation of learning from behavior.

### Standard tasks are limited in their ability to distinguish prioritized models

In standard signal-detection tasks in the psychosis literature, sensory statistics are often kept stable, so participants can perform well without learning. Furthermore, participants are usually rewarded for perceptual accuracy or are not explicitly rewarded for performance. So even if participants do learn, reward and sensory feedback are highly correlated which makes it challenging to determine if choices are driven by sensory or reward expectations. This dissociation is particularly important to evaluate theories that consider interactions between reward and sensory domains, like the hybrid bias model (Table 2). One approach to reveal the limitations of a task once we have a set of prioritized computational models is based on model simulations and recovery analyses that treat simulated data as a ground truth [18]. To distinguish between these models, at minimum key model parameters – the parameter(s) for a given model that drive increased high-confidence false alarms – must be recoverable in simulations. For example, to test initial bias models, participants’ initial expectations and learning rates for each domain (${\nu }_{S}^{t=0},{\nu }_{R}^{t=0},{\alpha }_{S},{\alpha }_{R}$) must be estimated. However, using a standard task, parameters are poorly recovered (Figure S6; Supplemental Methods).

### Designing a task that separates model variants

**Fig. 4:**
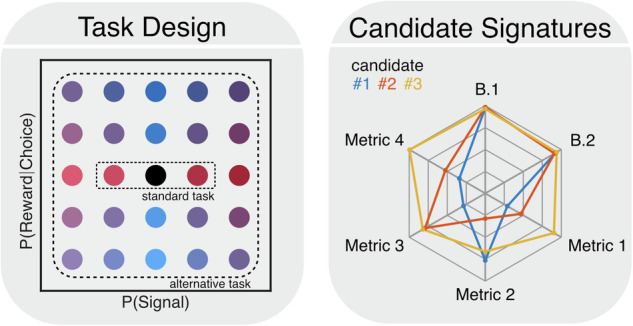
Dissociating prioritized hallucination models. Standard tasks rarely incentivize the development of both sensory and reward expectations which may be critical for determining relevant hallucination mechanisms. In contrast, an alternative task in which the probability of a signal and the probability of reward for a signal decision are systematically varied would incentivize learning of both sensory and reward expectations and the incorporation of those expectations into responses (left). This task also enables the isolation of qualitatively distinct behavioral signatures of each model which can faciliate falsification (right).

Given limitations of existing paradigms and to illustrate actionable next steps of our model prioritization scheme, we next set out to develop a novel task that would be better at empirically separating prioritized models. To address abovementioned concerns, we propose a task where the probability of a signal trial and the probability that a “yes” response yields additional reward are varied independently in a blocked structure (Fig. 4). Data from an ongoing study suggests that this task is feasible in human participants and show that task manipulations modulate behaviors in line with our model predictions [62].

This structure incentivizes simultaneous sensory and reward learning and decorrelates perceptual correctness from reward which should enable appropriate estimation of learning dynamics. If so, a recovery analysis should be able to demonstrate improvements, as our simulations show (Figure S7). Finally, a sufficiently rich task design should allow different models to make *qualitatively* distinct or even opposing predictions apparent in model-agnostic analyses of raw behavioral data. For example, the asymmetric sensory learning and initial sensory bias models make opposing predictions about how signal probability influences the evolution of perceptual confidence over the course of a block (Figure S8). By combining several of these metrics, unique ‘fingerprints’ of these models can be identified and models that do not exhibit signatures observed in the data can be falsified (Fig. 4; Figure S8).

In sum, once a set of models have been prioritized, we recommend testing them using a paradigm that emphasizes their unique signatures [18]. Our simulations suggest that existing paradigms fall short of this goal, but that computational models can be used to design new experiments that isolate the unique model signatures and rule out models inconsistent with empirical results.

## Discussion

Exciting advances in the development of hallucination theories have taken place in recent years [1–3, 5, 6, 8–13, 36]. Several distinct mechanistic hypotheses of hallucinations are under active investigation, but progress in arbitrating between them has been limited by challenging operationalizations of some hypotheses and a dearth of experimental frameworks designed to directly compare and falsify them. Falsification is particularly important given the continued appeal of influential theories like ‘aberrant salience’ [7, 8]. The face value of this hypothesis has made it a popularly invoked explanation for a wide array of disparate results [107]. Critically, our intention is not to question the value of this hypothesis or the evidence supporting altered reward processing in psychosis. Indeed, one of our prioritized models (hybrid bias) is broadly consistent with the ‘aberrant salience’ hypothesis. In more general terms, we argue that it seems counterproductive to continue debating theories that do not map onto a unique circuit computation (Fig. 1), and that it may be more fruitful to test sets of specific operationalizations of these theories that do.

To this end, here we described a step-by-step strategy for specifying, prioritizing, and testing hallucination theories. We first developed a constrained model of decision-making which provided a base architecture to formulate a series of potential models of hallucinations (Fig. 2). This architecture was informed by generally accepted models of reward learning and perceptual decision-making which have been extensively validated by convergent translational data [13, 22, 33, 35, 51, 62]. While there are other ways to instantiate such a model (e.g., alternative learning rules), predictions would likely be qualitatively similar (see Figure S5). We then prioritized models that capture key empirical data in the hallucination literature (Fig. 3). Since these prioritized models each map onto a unique circuit computation, we see them as a starting point for a new, falsifiable taxonomy of mechanistic hallucination theories (Table 2). This taxonomy can be used to identify the most promising subtypes of prominent theories in the field. For example, our simulations suggest that the initial sensory bias model should be favored over the slow sensory learning model to operationalize the ‘strong prior’ hypothesis. Importantly, this general pipeline can be readily extended to operationalize other leading cognitive (e.g., related to volatility [12, 28] and other aspects of inference [1, 20, 108]) and neurobiological (e.g., the role of acetylcholine in modulating top-down predictions [88, 109]) hypotheses in the literature. Finally, we demonstrated that a key strength of prioritizing models in this manner is that it enables the design of experiments that emphasize their qualitative differences, which promotes model refinement and falsification [16, 18]. Moreover, since these experimental paradigms are optimized to test multiple hypotheses simultaneously, they avoid reliance on a single a priori hypothesis and are well-suited to uncover distinct mechanisms for hallucinations across subpopulations.

Overall, we believe that progress in our mechanistic understanding of hallucinations will require testing a broader set of model families selected in a principled manner together with a carefully defined criteria for falsification. Identifying a more precise underlying model with a distinct neurobiological substrate could ultimately facilitate the development of more targeted treatments with increased efficacy and tolerability that improve patients’ quality of life.

## Supplementary information

Supplementary Material
