# Supplementary material for: Refining mechanistic models of hallucinations for enhanced translatability
Source: Transl Psychiatry. 2025 Dec 3;16:31. doi: 10.1038/s41398-025-03773-x (PMC12811618; doi:10.1038/s41398-025-03773-x)
Supplement: Supplementary file 1 — Supplementary Material [file 41398_2025_3773_MOESM1_ESM.docx]

# **SUPPLEMENTAL METHODS**

## Decision-Making Model

## *Comparing Model Expectation Learning to ‘Strong-Prior’ Hypotheses*

According to ‘strong-prior’ hypotheses, patients with psychosis overweight their prior beliefs relative to new evidence^1^. These theories can be formulated in terms of a sequential belief updating process that involves deliberating between two complementary hypotheses. Under Bayesian inference the posterior probability of experiencing a signal relative to noise given a sensory observation $s$ is the product of prior expectations and the likelihood of the observation:

| $\frac{P\left( \left. \mathrm{signal} \right\vert s \right)}{P\left( \left. \mathrm{noise} \right\vert s \right)}=\frac{P\left( \mathrm{signal} \right)}{P\left( \mathrm{noise} \right)}\cdot\frac{P\left( \left. s \right\vert\mathrm{signal} \right)}{P\left( \left. s \right\vert\mathrm{noise} \right)}$ | *Eq. S1* |
| --- | --- |

which can be represented in log-odds (logit) space for a convenient representation of evidence integration:

| $\log\frac{P\left( \left. \mathrm{signal} \right\vert s \right)}{P\left( \left. \mathrm{noise} \right\vert s \right)}=\log\frac{P\left( \mathrm{signal} \right)}{P\left( \mathrm{noise} \right)}+\log\frac{P\left( \left. s \right\vert\mathrm{signal} \right)}{P\left( \left. s \right\vert\mathrm{noise} \right)}$ | *Eq. S2* |
| --- | --- |

or simply

| $\mathrm{Posterior}_{t}=\mathrm{Prior}_{t}+\mathrm{Likelihood}_{t}$ | *Eq. S3* |
| --- | --- |

A common parameterization of this update rule applies a multiplicative weight $\omega_{1}$ to the prior expectation and a multiplicative weight $\omega_{2}$ to the likelihood^2,3^:

| $\mathrm{Posterior}_{t}={\omega_{1}\cdot Prior}_{t}+{\omega_{2}\cdot Likelihood}_{t}$ | *Eq. S4* |
| --- | --- |

Larger values of $\omega_{1}$, which have been shown to correlate with psychosis severity^2^, suggest that observers’ prior expectations more strongly influence their posterior. A simplified version of this model assumes a tradeoff between the influence of prior expectations and likelihood on posterior beliefs:

| $\mathrm{Posterior}_{t}={\omega\cdot Prior}_{t}+{(1-\omega)\cdot Likelihood}_{t}$ | *Eq. S5* |
| --- | --- |

In this formulation, a ‘strong prior’ would be consistent with a large $\omega$ value since observers preferentially incorporate their prior expectations into their posteriors.

Our model updates expectations $\nu$ by comparing the outcome $O$ of the current trial (either reward or stimulus information) to their subjective confidence $C$ to generate a prediction error (Eqs. 7 & 9) that is then scaled by a learning rate $\alpha$ (Eqs. 8 & 10):

| $\nu^{t+1}=\nu^{t}+\alpha\cdot\left( O^{t}-C^{t} \right)$ | *Eq. S6* |
| --- | --- |

Using our definitions of confidence (Eqs. 4-6), this formula can be rewritten as:

| $\nu^{t+1}=\left( 1-\alpha\right)\cdot\nu^{t}+\alpha\cdot O^{t}$ | *Eq. S7* |
| --- | --- |

This formula has the same structure as the belief update described above (Eq. S5), with an inverse relationship between $\omega$ and $\alpha$. Therefore we consider a reduced value for $\alpha$ to approximate the ‘strong prior’ hypothesis.

## *Comparing Model Expectation Learning to Decision Criterion Updating*

We describe an observer that makes the decision $\theta$ (“Yes” = 1, “No” = 0) with the highest expected value on each trial (Eq. 3). This process can also be equivalently framed as comparing sensory evidence $\mu_{Lik}$ on a given trial to an internal decision criterion $\beta$:

| $\theta^{t}= \left\{ \begin{matrix} 1, & \mathrm{if} \beta\geq\mathrm{logit} \left( \mu_{Lik}^{t} \right) \\ 0, & \mathrm{otherwise} \end{matrix} \right.$ | *Eq. S8* |
| --- | --- |

An ideal observer sets their decision criterion at the point where the expected value of both decisions given a sensory observation $s$ is equal^4,5^:

| $\mathbb{E}\left[ \left. r(\theta_{\mathrm{signal}}) \right\vert s \right]\mathbb{=E}\left[ \left. r(\theta_{\mathrm{noise}}) \right\vert s \right]$ | *Eq. S9* |
| --- | --- |

where

| $\mathbb{E}\left[ \left. r(\theta_{\mathrm{signal}}) \right\vert s \right]= P\left( \left. \mathrm{signal} \right\vert s \right)\cdot r\left( \mathrm{Hit} \right)+P\left( \left. \mathrm{noise} \right\vert s \right)\cdot r\left( \mathrm{FA} \right)$ | *Eq. S10* |
| --- | --- |
|  |  |
| $\mathbb{E}\left[ \left. r(\theta_{\mathrm{noise}}) \right\vert s \right]= P\left( \left. \mathrm{signal} \right\vert s \right)\cdot r\left( \mathrm{Miss} \right)+P\left( \left. \mathrm{noise} \right\vert s \right)\cdot r\left( \mathrm{CR} \right)$ | *Eq. S11* |

Using Bayes rule^4,5^ we can show that the optimal criterion is determined by perceptual and reward expectations:

| $\beta=\log\frac{P(signal)}{P(noise)}+\log\frac{r\left( \mathrm{Hit} \right)-r(\mathrm{Miss})}{r\left( \mathrm{CR} \right)-r(\mathrm{FA})}$ | *Eq. S12* |
| --- | --- |

Thus, since these quantities are learned through experience in our model, this learning can also be framed as updating a decision criterion.

## *Prior-Based Sensory Learning Rule*

Following previous work^6^, in our decision-making model sensory prediction errors are computed as the difference between perceptual confidence and the sensory outcome (Eq. 9). However, observers could also compute a prediction error as the difference between the sensory outcome and sensory expectation:

| $\delta_{S}^{t}=\left( F^{t}-\nu_{S}^{t} \right)$ | *Eq. S13* |
| --- | --- |

We highlight this alternative rule because sensory expectations learned in this manner are particularly challenging to estimate independently from reward expectations using standard signal detection tasks (Figure S5). This motivates the use of a more robust task that can separate these learning processes regardless of the assumed learning rule (Figure S6).

**Cognitive Tasks**

## *Standard Task*

Following common signal detection task designs in the literature^6,7^, here we consider a ‘standard’ task to have a base rate of 50% signal trials. The loudness of a signal presentation (e.g., signal to noise ratio) is selected randomly to have a detection probability of 25%, 50%, or 75% (defined as the likelihood $\mu_{Lik}$ in the model). Importantly, there is no higher-level statistical structure to the sensory environment (e.g., signal base rate manipulation) that observers could learn to improve performance. Additionally, perceptually correct responses are rewarded equally regardless of choice, and trials are presented in sequence without a defined block structure.

## *Proposed Task*

In our example proposed task, signal base rate (i.e., probability of a signal trial) and reward probability (i.e., probability of a “Yes” response yielding additional reward) vary independently in a blocked structure. This serves to partially decorrelate perceptual correctness from reward receipt while incentivizing learning of both perceptual and reward contingencies. Furthermore, by resetting observers’ expectations at the beginning of each block (i.e., informing observers that underlying contingencies may change), the dynamics of the learning process can be well estimated.

**Simulations**

For both tasks, we simulate 240 trials for each observer. In the *standard task*, these 240 trials are part of a single block. In the *proposed task*, we simulate 12 blocks of 20 trials each. There are three categories of blocks: signal, reward, and hybrid manipulation blocks. In signal blocks, reward probability is fixed at 50% and signal probability is either 10%, 30%, 70%, or 90% (4 blocks total). In reward blocks, signal probability is fixed at 50% and reward probability either 10%, 30%, 70%, or 90% (4 blocks total). In hybrid blocks, signal and reward probability are either 20% or 80% (all combinations result in 4 blocks total).

For both tasks, at the beginning of blocks expectations are set to observers’ respective $\nu^{t=0}$. In the *standard task*, since there is only one block, expectations start at $\nu^{t=0}$ and are never reset.

## *Assessing Candidate Models*

To evaluate candidate models (Figure 3; Table 2) we simulated observers on a standard signal detection task. The block structure was held constant across observers, but trial-by-trial stimuli and rewards were randomized. To be considered a prioritized hallucination model, a cognitive alteration should satisfy group B constraints. Specifically, we expect that as the magnitude of a parameter alteration increases, there will be a concomitant increase in signal decision bias (measured by choice ratio: $\frac{False Alarm Rate}{Miss Rate}$ ) and false alarm confidence (measured by decision confidence $C_{D}^{t}$ on false alarm trials; Eq. 6).

We simulated 100 observers for each model with a range of alteration magnitudes. For example, slow sensory learning observers had fixed reward learning ($\alpha_{R}$) and varied sensory learning ($\alpha_{S}\in\{0, 0.001, 0.002, 0.003,\ldots,0.098, 0.099, 0.1\}$). Rank correlations between bias magnitude, choice ratio, and false alarm confidence were then computed. Constraints were satisfied if there was a robust, monotonically increasing relationship between alteration magnitude (in this example reduced $\alpha_{s}$) and *both* choice ratio and false alarm confidence.

## *Parameter Recovery*

To determine whether a given participant’s data is consistent with a particular cognitive alteration (e.g., initial reward bias vs asymmetric perceptual) using model-based approaches, parameters must be reliably estimable from task data. We evaluate whether distinct parameter values in our model can be identified in standard tasks and proposed tasks using parameter recovery^8^. By comparing simulated, ‘ground-truth’ parameters to parameters inferred from the simulated data we can assess whether the conditions of the task allow for sufficient behavioral variability to measure each model parameter. We randomly selected parameters for 100 observers and simulated each observer on both task types. We then fit the simulated data (using Matlab’s *fmincon*) to our model and compared the ground truth stimulated parameters to recovered parameters.

#
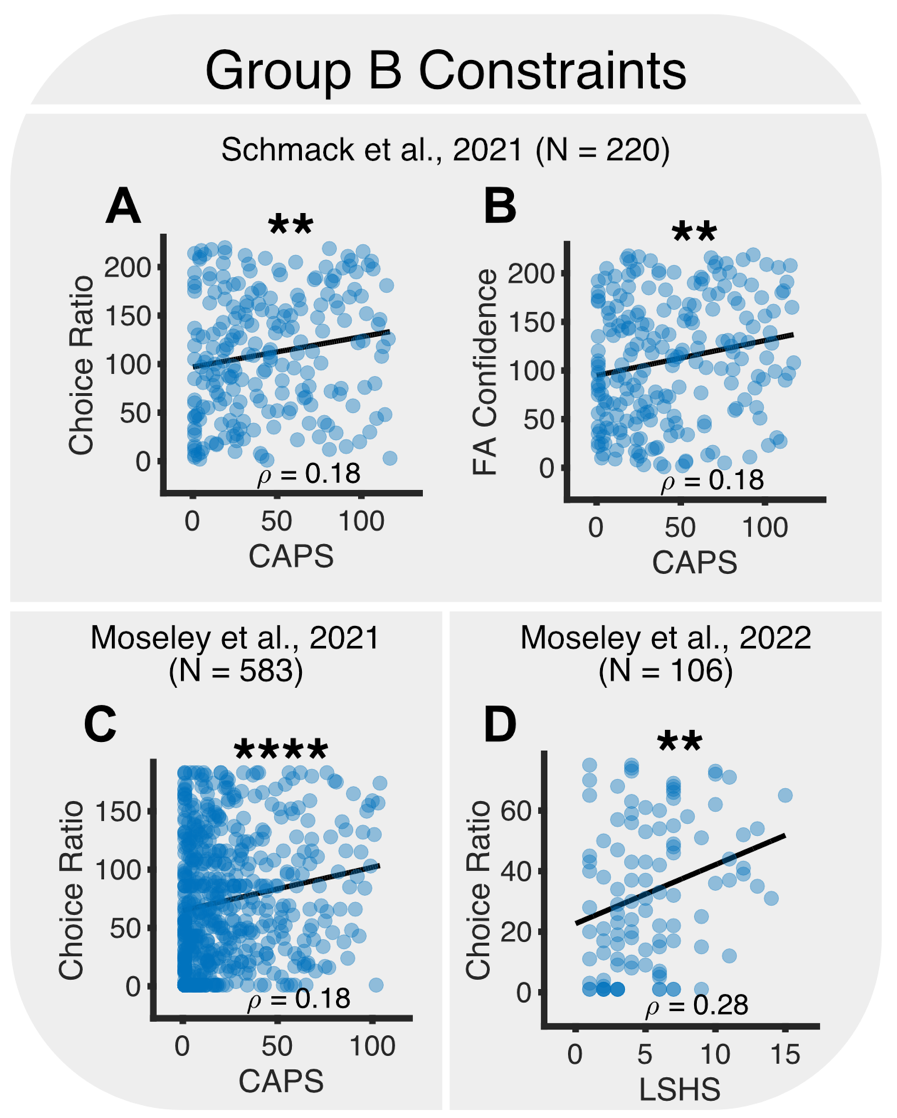
**SUPPLEMENTAL FIGURES**

**Figure S1. Group B Constraints in Open-Source Datasets.** Hallucination proneness is positively correlated with choice ratio (A, C, and D) and false alarm confidence (B) in large, open-source datasets. For visualization, ranked metrics are plotted. Correlations represent Spearman’s rho.

CAPS=Cardiff Anomalous Perceptions Scale; LSHS=Launey-Slade Hallucination Scale; **=p<0.01, ****=p<0.0001


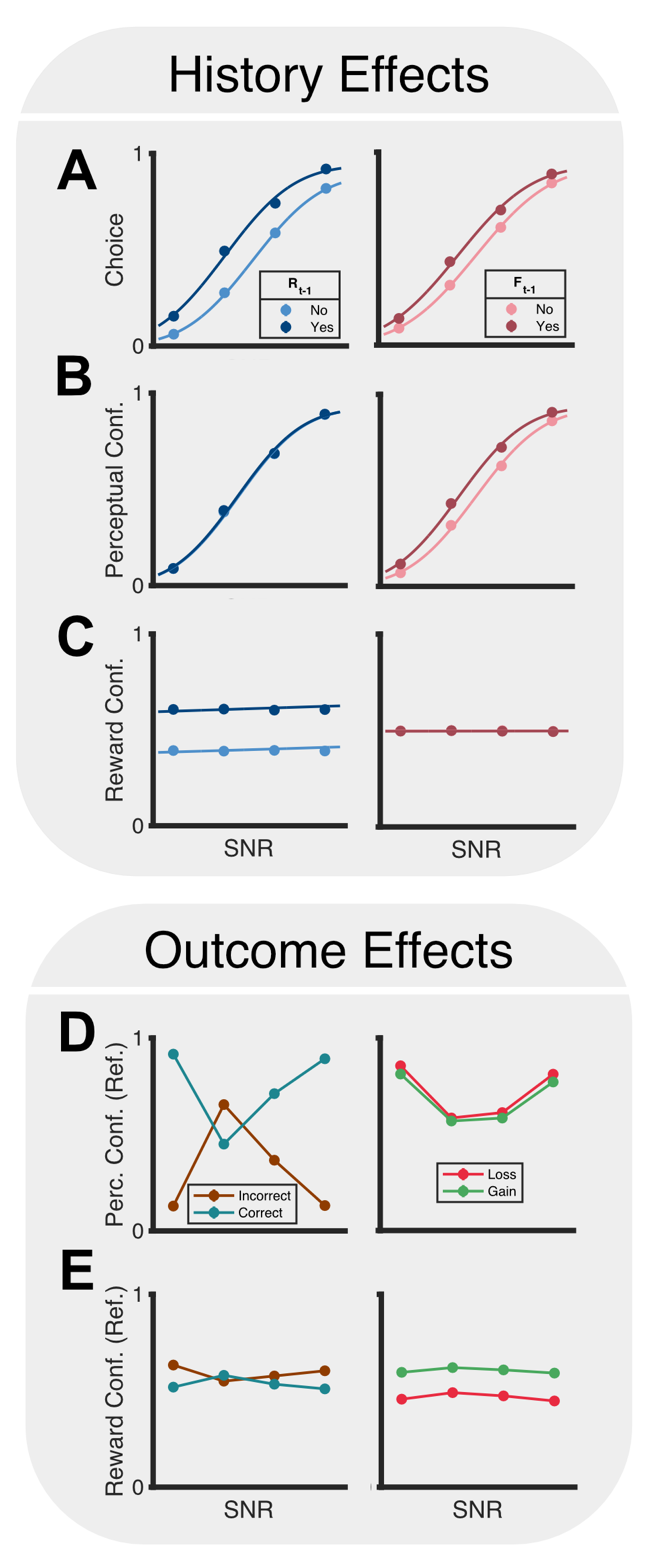


**Figure S2. Simulating the Decision-Making Model.**  Simulations from the model show expected history dependencies where previous trial evidence that ‘Yes’ decisions are more valuable (dark blue) bias choice (A) and reward confidence (C) but not perceptual confidence (B). Analogously, previous signal trials (dark red) bias choice and perceptual confidence but not reward confidence. Simulations also show expected outcome dependencies where perceptual confidence is higher on perceptually accurate trials and this effect scales with difficulty (i.e., SNR, D). Analogously, reward confidence is higher on trials when reward is received and is not sensitive to SNR (E). Note that for outcome effects plotted confidence reports are referenced to their respective outcomes (0=certain of negative outcome, 1=certain of positive outcome).


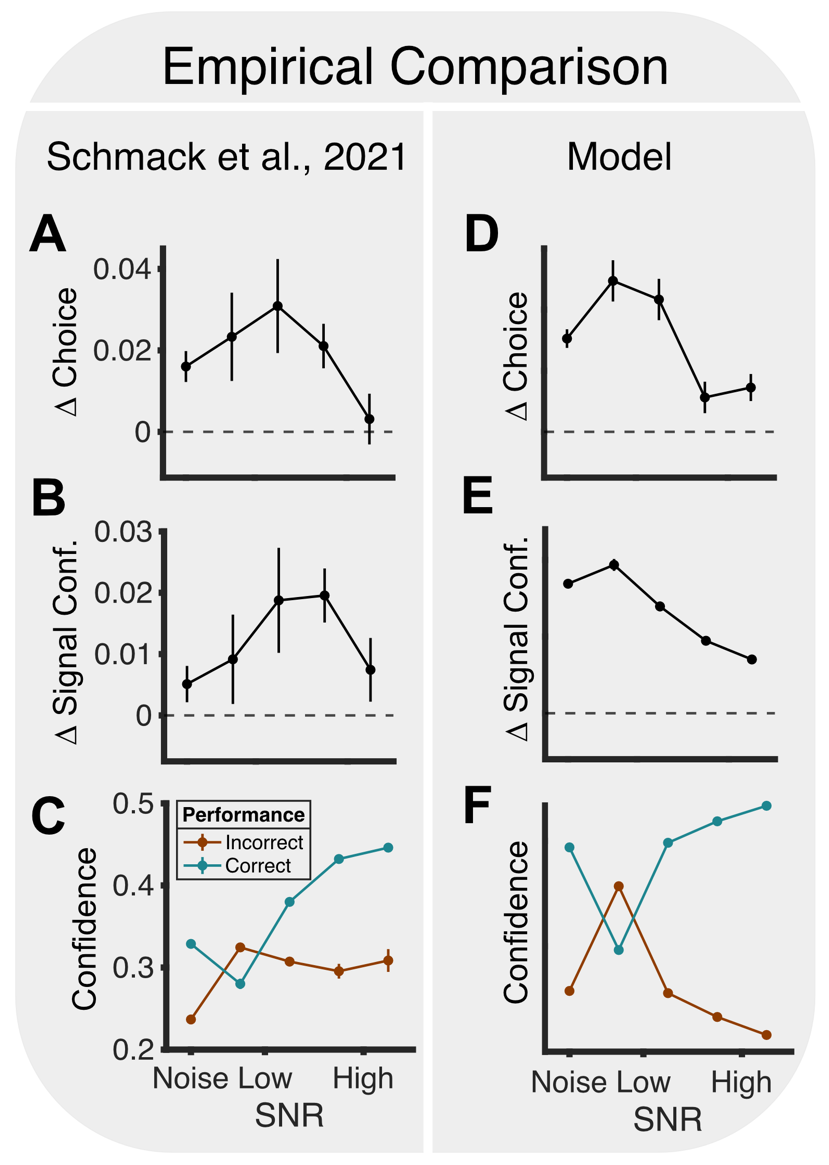


**Figure S3. Comparing Model Predictions to Real Data.**  Participants report more “Yes” choices (stimulus history: t=9.98, p<0.0001, logistic mixed effects regression) and higher signal confidence (stimulus history: t=43.79, p<0.0001, linear mixed effects regression) when more signal trials were experienced recently. For visualization, we show the difference in “Yes” choice rates (A) and signal confidence reports (B) following two consecutive signal trials (i.e., F_t-1_=1 and F_t-2_=1) versus two consecutive noise trials (i.e. F_t-1_=0 and F_t-2_=0). Simulated model observers with a modest learning rate ($\alpha=0.05$) show similar history effects for both choice (D) and signal confidence (E). Participants also display higher confidence on correct trials and this effect scales with SNR (C; performance*SNR: t=203.24, p<0.0001, linear mixed effects regression on signal trials). Simulated observers show a similar pattern of confidence (F). Error bars represent standard error of the mean across subjects.


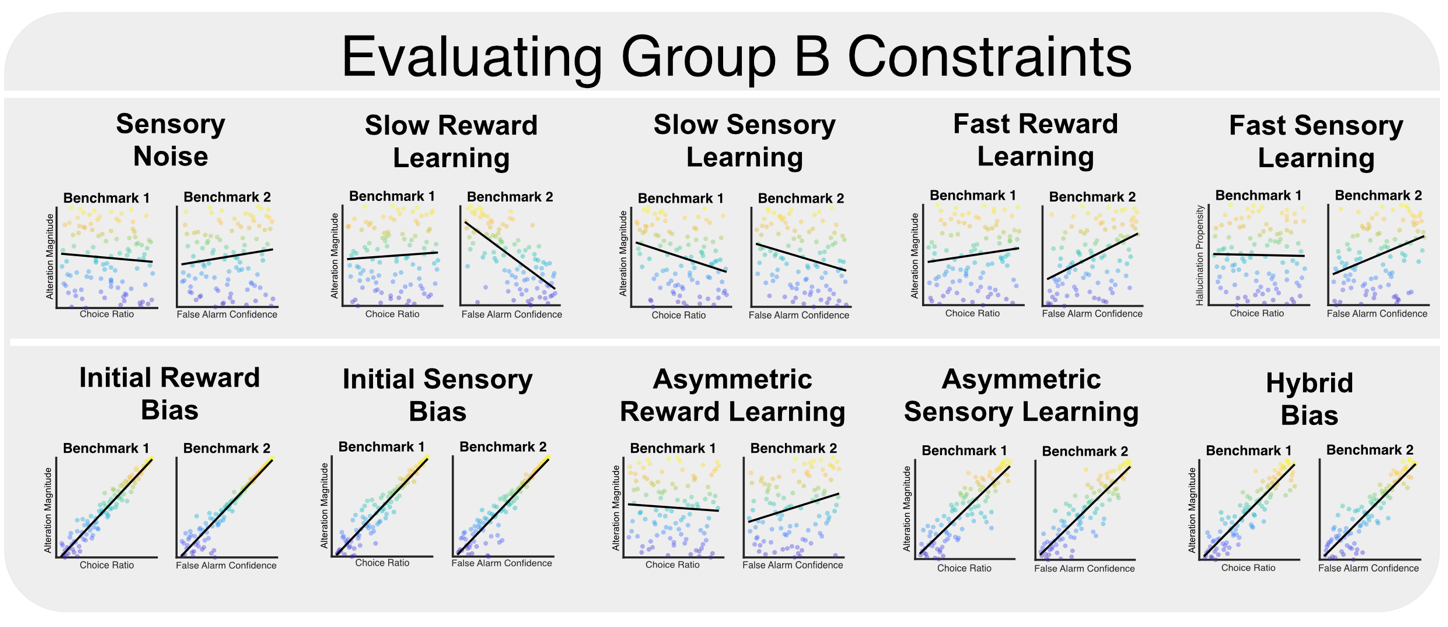


**Figure S4. Evaluating Group B Constraints in Confidence-Based Learning Model.** To prioritize candidate hallucination models, we evaluate whether a model can explain well-replicated results in the literature of hallucination-like phenomena. Specifically, we simulate observers with parameter alterations (see Supplemental Methods) and evaluate whether a given alteration systematically drives an increase in choice ratio (Constraint B.1) and false alarm confidence (Constraint B.2) in a signal detection task (Table 2). Alterations that satisfy both constraints are considered priorities. All simulations were generated from a model with a confidence-based learning rule (Eq. 9).


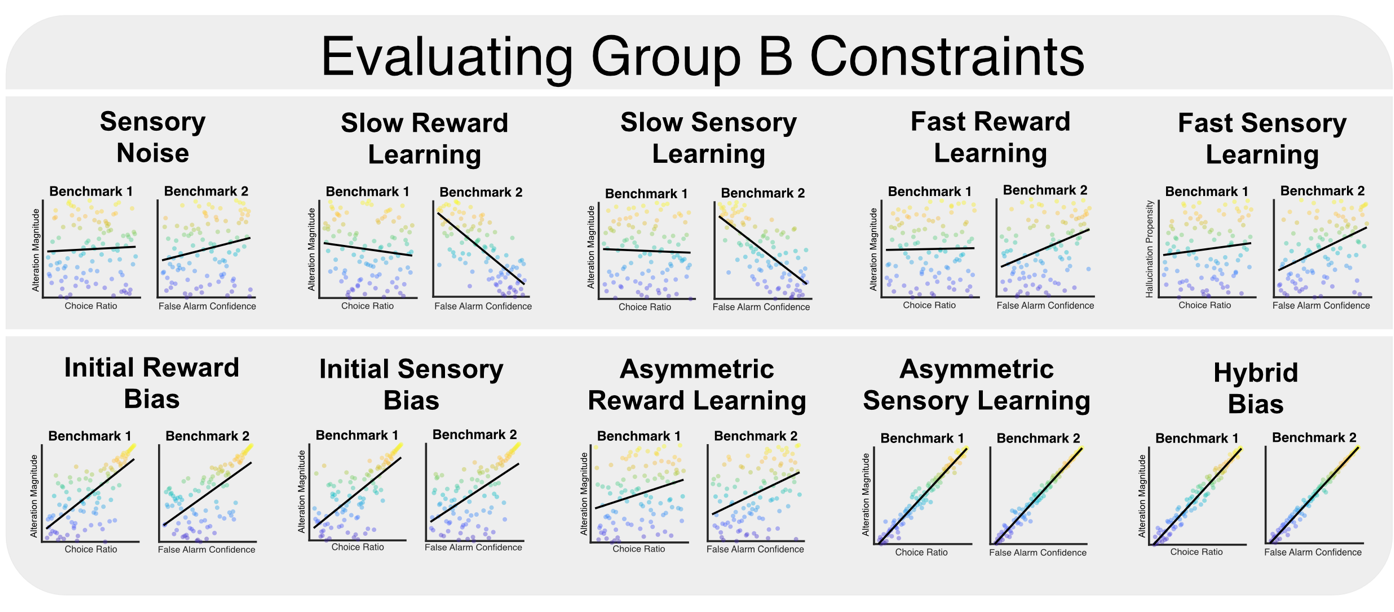

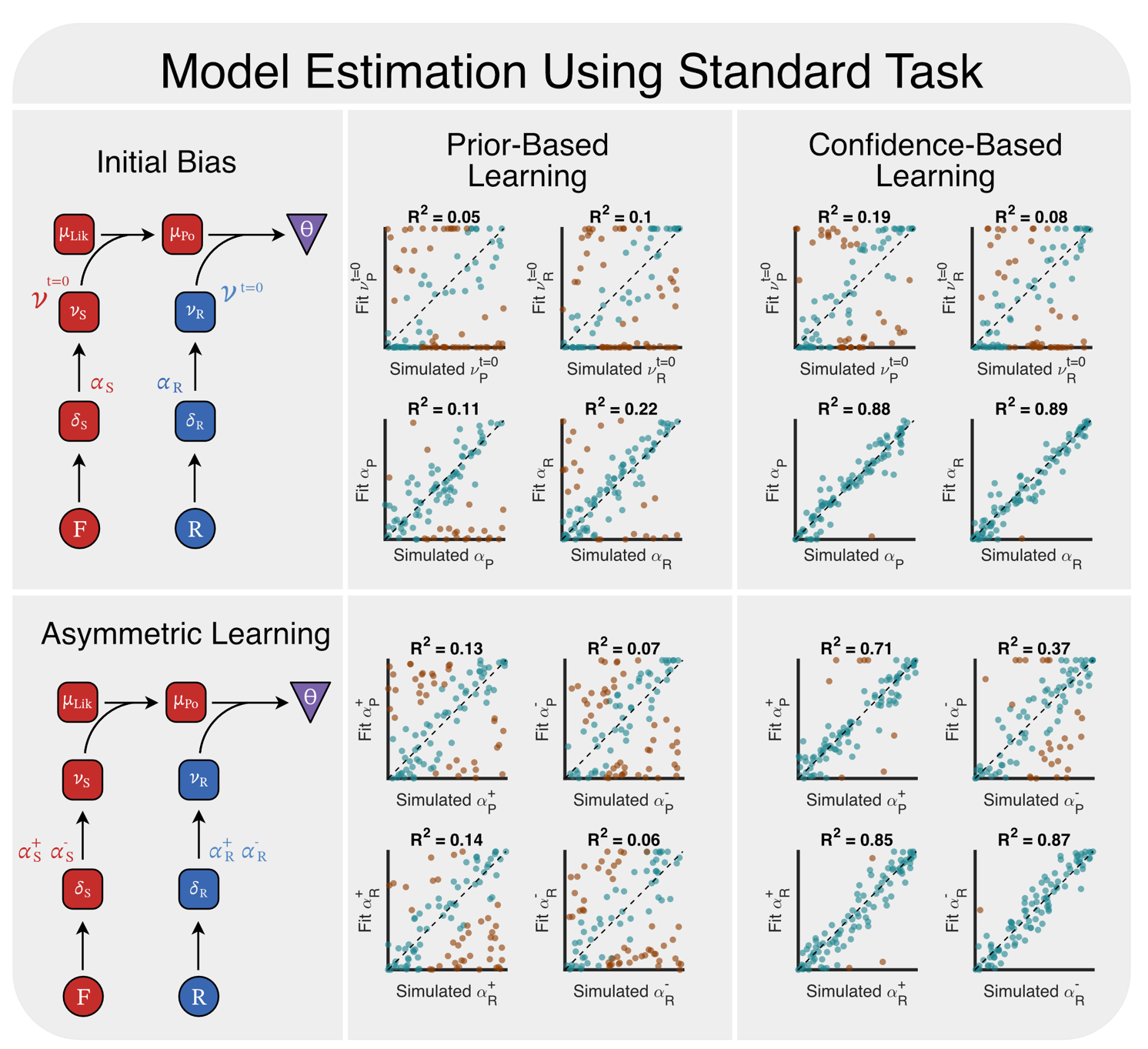


**Figure S5. Evaluating Group B Constraints in Prior-Based Learning Model.** Observers simulated as in Figure S4 but from a model with a prior-based learning rule (Eq. S13).

**Figure S6. Parameter Estimation Using Standard Signal Detection Task.** Standard signal detection tasks rarely incentivize the learning of the statistical structure of the environment (e.g., sensory and/or reward probabilities). Additionally, participants are often rewarded for perceptual correctness regardless of choice. Since we have a set of prioritized computational models, we can evaluate the richness of this task using model simulations and recovery analyses that treat simulated data as ground truth. To be able to distinguish between models of interest, at minimum key model parameters – the parameter(s) for a given model that drive increased high-confidence false alarms – must be recoverable in simulations. For example, to test initial bias models (Top), participants’ initial expectations and learning rates for each domain ($\nu_{P}^{t=0},\nu_{R}^{t=0},\alpha_{P},\alpha_{R}$) must be estimated. To test asymmetric learning models (Bottom), participants’ positive and negative learning rates for each domain must be estimated ($\alpha_{S}^{+},\alpha_{S}^{-},\alpha_{R}^{+},\alpha_{R}^{-}$). Both initial bias and asymmetric learning models show poor recovery when using a prior-based sensory learning rule (Left, Eq. S13). When using a confidence-based learning rule (Right, Eq. 9), the initial bias model is also recovered poorly while the asymmetric learning model shows reasonable recovery.


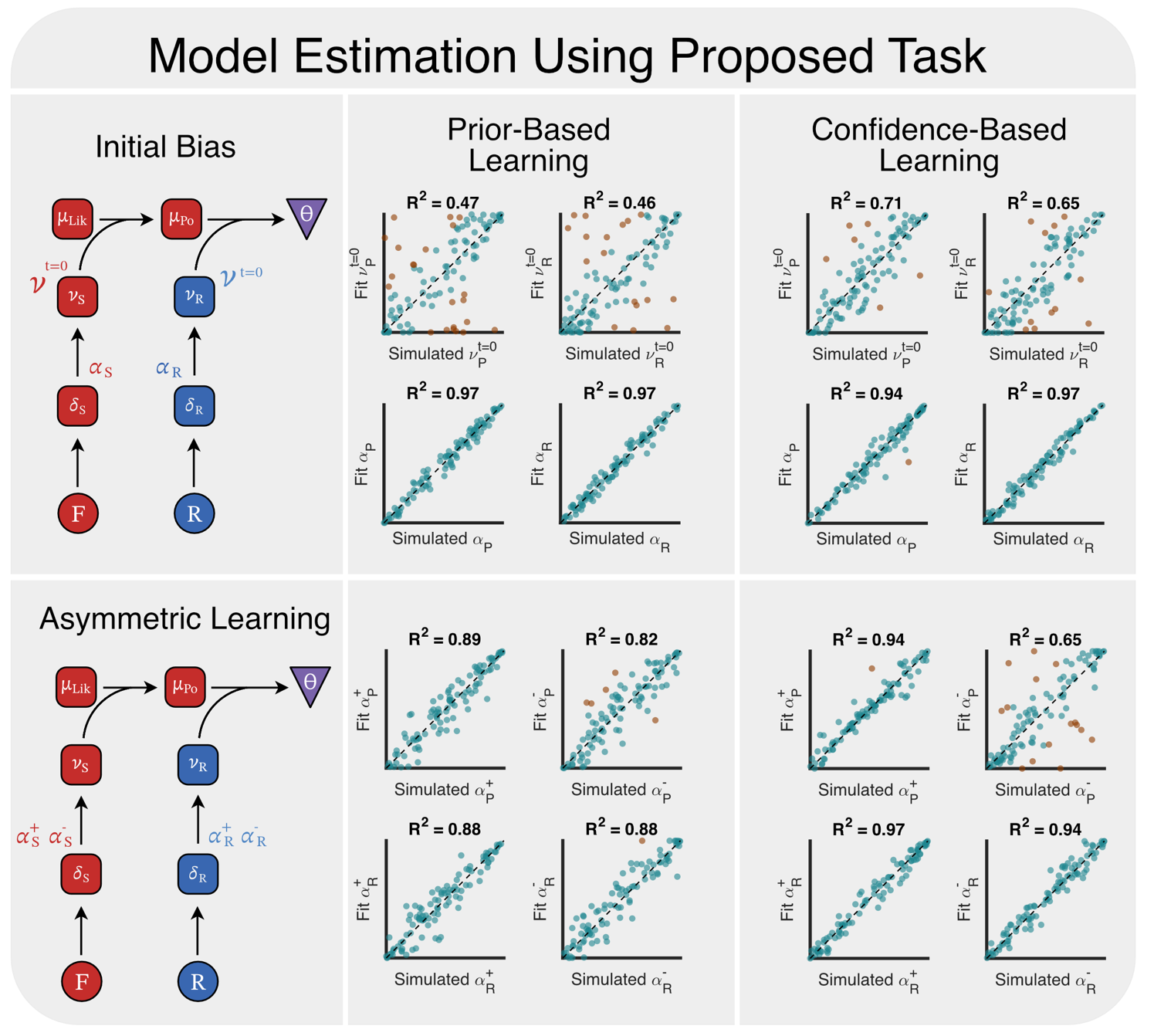


**Figure S7. Parameter Estimation Using Proposed Signal Detection Task.** In the proposed task, signal base rate and reward probability vary independently in a blocked structure. This structure decorrelates perceptual correctness from reward and incentivizes learning of both contingencies. The blocked structure also allows for more accurate estimation of learning trajectories. Using the same approach described in Figure S5, we find that regardless of the learning rule, both the initial bias and asymmetric learning models are well recovered.


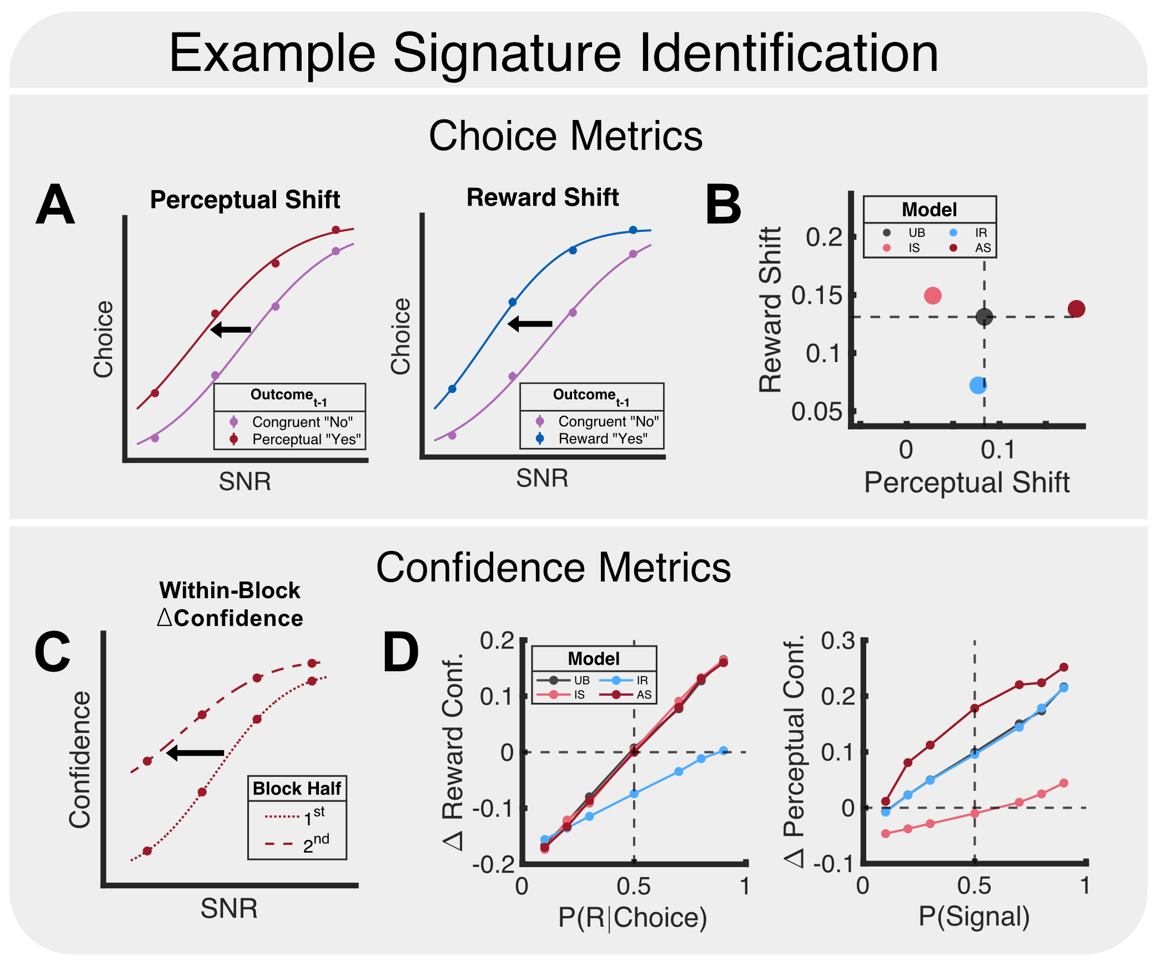


**Figure S8. Example Metrics to Identify Prioritized Hallucination Model Signatures.** By comparing trials with different recent outcomes, qualitatively distinct patterns of perceptual and reward learning can be identified. For example, on a trial where an observer correctly reports that there was no signal present and their response is rewarded, learning in both domains should promote future “No” responses (i.e., perceptual “No” and reward “No” [pNrN]). In contrast, on a trial where an observer correctly reports that a signal was present, but their response is not rewarded, learning in the sensory domain promotes future “Yes” responses while learning in the reward domain promotes future “No” responses (i.e., perceptual “Yes” and reward “No” [pYrN]). Comparing choices on trials following these two trial types will specifically isolate a model’s sensory history effects, which we term a ‘perceptual shift’ (A, left). An analogous ‘reward shift’ can be computed to isolate a model’s reward history effects (A, right). All models show positive perceptual and reward shifts since they learn in both domains, but as bias magnitude increases, perceptual and reward shifts become smaller for initial sensory and initial reward models, respectively. In contrast, perceptual shift becomes larger for the asymmetric sensory model (B). Confidence trajectories within blocks can also be used to assess model signatures. To summarize these trajectories, we compute the difference between average confidence in the first and second halves of each block (which we term ‘within-block ∆ confidence’; C). All models show a positive slope between their within-block confidence shifts and the corresponding block probabilities (D). However, models with initial biases show a slope reduction as a function of bias magnitude specifically in the confidence domain corresponding with their alteration (i.e. initial sensory bias models show a reduction in their perceptual confidence slope but not their reward confidence slope). In contrast, models with asymmetric learning show an intercept increase as a function of bias magnitude.

UB=unbiased; IR=Initial reward; IS=Initial sensory; AS=Asymmetric sensory

# **SUPPLEMENTAL REFERENCES**

1. Horga, G. & Abi-Dargham, A. An integrative framework for perceptual disturbances in psychosis. *Nat Rev Neurosci* **20**, 763–778 (2019).

2. Baker, S. C., Konova, A. B., Daw, N. D. & Horga, G. A distinct inferential mechanism for delusions in schizophrenia. *Brain* **142**, 1797–1812 (2019).

3. Ashinoff, B. K., Buck, J., Woodford, M. & Horga, G. The effects of base rate neglect on sequential belief updating and real-world beliefs. *PLOS Computational Biology* **18**, e1010796 (2022).

4. Dayan, P. & Daw, N. D. Decision theory, reinforcement learning, and the brain. *Cognitive, Affective, & Behavioral Neuroscience* **8**, 429–453 (2008).

5. Wickens, T. D. *Elementary Signal Detection Theory*. xiii, 262 (Oxford University Press, New York, NY, US, 2002).

6. Schmack, K., Bosc, M., Ott, T., Sturgill, J. F. & Kepecs, A. Striatal dopamine mediates hallucination-like perception in mice. *Science* **372**, eabf4740 (2021).

7. Moseley, P. *et al.* Correlates of Hallucinatory Experiences in the General Population: An International Multisite Replication Study. *Psychol Sci* **32**, 1024–1037 (2021).

8. Wilson, R. C. & Collins, A. G. Ten simple rules for the computational modeling of behavioral data. *eLife* **8**, e49547 (2019).
